# Comparison of CT derived body composition at the thoracic T4 and T12 with lumbar L3 vertebral levels and their utility in patients with rectal cancer

**DOI:** 10.1186/s12885-023-10522-0

**Published:** 2023-01-16

**Authors:** Aisha A Arayne, Richard Gartrell, Jing Qiao, Paul N Baird, Justin MC Yeung

**Affiliations:** 1 Department of Surgery, Western Health, Footscray, VIC Australia; 2 Department of Surgery, Western Precinct, University of Melbourne, VIC, Australia; 3 Department of Colorectal Surgery, Western Health, Footscray, VIC Australia; 4 Department of Surgery, Ophthalmology, University of Melbourne, Victoria, Australia; 5 Western Chronic Disease Alliance, Western Health, Sunshine, VIC Australia; 6 Department of Surgery, Melbourne Medical School – Western Health, Faculty of Medicine, Dentistry and Health Sciences, The University of Melbourne, Level 3, WCHRE Building, Sunshine Hospital, 176 Furlong Road, St Albans, VIC 3021 Australia

**Keywords:** Skeletal muscle mass, L3, T4, T12, Computed tomography, Sarcopenia

## Abstract

**Background:**

Computed tomography (CT) derived body composition measurements of sarcopenia are an emerging form of prognostication in many disease processes. Although the L3 vertebral level is commonly used to measure skeletal muscle mass, other studies have suggested the utilisation of other segments. This study was performed to assess the variation and reproducibility of skeletal muscle mass at vertebral levels T4, T12 and L3 in pre-operative rectal cancer patients. If thoracic measurements were equivalent to those at L3, it will allow for body composition comparisons in a larger range of cancers where lumbar CT images are not routinely measured.

**Research methods:**

Patients with stage I – III rectal cancer, undergoing curative resection from 2010 – 2014, were assessed. CT based quantification of skeletal muscle was used to determine skeletal muscle cross sectional area (CSA) and skeletal muscle index (SMI). Systematic differences between the measurements at L3 with T4 and T12 vertebral levels were evaluated by percentile rank differences to assess distribution of differences and ordinary least product regression (OLP) to detect and distinguish fixed and proportional bias.

**Results:**

Eighty eligible adult patients were included. Distribution of differences between T12 SMI and L3 SMI were more marked than differences between T4 SMI and L3 SMI. There was no fix or proportional bias with T4 SMI, but proportional bias was detected with T12 SMI measurements. T4 CSA duplicate measurements had higher test–retest reliability: coefficient of repeatability was 34.10 cm^2^ for T4 CSA *vs* 76.00 cm^2^ for T12 CSA. Annotation time (minutes) with L3 as reference, the median difference was 0.85 for T4 measurements and -0.03 for T12 measurements. Thirty-seven patients (46%) had evidence of sarcopenia at the L3 vertebral level, with males exhibiting higher rates of sarcopenia. However, there was no association between sarcopenia and post-operative complications, recurrence or hospital LOS (length of stay) in patients undergoing curative resection.

**Conclusions:**

Quantifying skeletal muscle mass at the T4 vertebral level is comparable to measures achieved at L3 in patients with rectal cancer, notwithstanding annotation time for T4 measurements are longer.

## Introduction

Loss of muscle mass and function is an age-related pathological process and in extreme cases is referred to as sarcopenia, which is associated with decreased survival in cancer patients [1–3]. As body composition measurements of sarcopenia can serve as markers of overall health, they may therefore play an important role in the prognostication of disease processes. The third lumbar vertebral level (L3) gold standard level for body composition assessment in rectal cancer patients, but for other cancer types, thoracic CT slices have been used for body composition analysis as their CT imaging may not include slices from the lumbar region [4]. In order to compare the effect of body composition changes between different cancers, it is vital that we have the ability to adequately utilise the same vertebral level in this comparison. Body composition is almost exclusively performed opportunistically using CT scans acquired as part of routine care. There is unfortunately very little information within the literature which had compared the reproducibility and ease of measurement between lumbar and thoracic CT slices.

Our primary objective was therefore to assess the variation in skeletal muscle mass at thoracic 4, thoracic 12 and lumbar 3 regions in patients with colorectal cancer (CRC) prior to surgery. Using L3 measurements as a reference, we chose to compare the body composition measurements and patient clinical outcomes at the fourth and twelfth thoracic vertebral levels (T4, T12), as these are two of the commonly utilised thoracic levels in the literature [5–7]. Our secondary objective was to determine the repeatability and measurement time of body composition assessments at these three vertebral levels.

## Methods

The study was performed according to the Helsinki declaration, the International Conference on Harmonisation Guidelines for Good Clinical Practice and approved by the local institutional ethics committee. Quality Assurance Project Number: Quality Assurance 2020.24 ERM ID Reference Number: 63907.

**Fig. 1:**
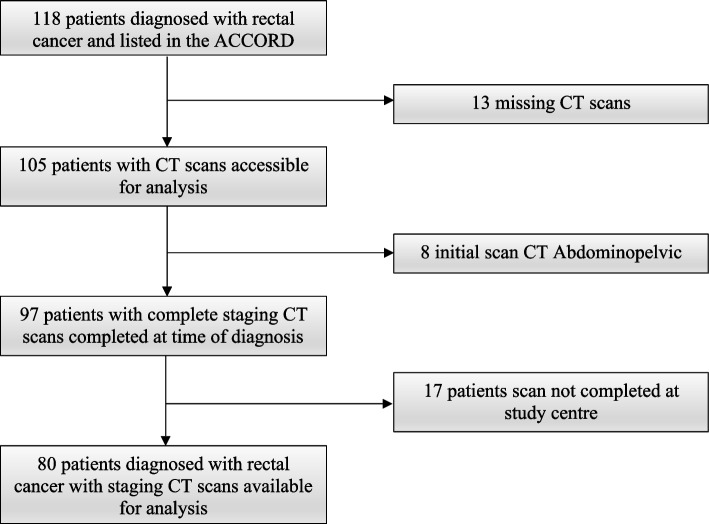
Flow-chart. 80 of 118 patients from Australian Comprehensive Cancer Outcomes Research Database were eligible for analysis

A retrospective analysis was performed on rectal cancer patients from a pre-existing prospectively maintained database (ACCORD). Patients were identified over a 5-year period between January 2010 to December 2014 from a single tertiary centre, Footscray Hospital, Western Health, Melbourne, Australia. The following inclusion criteria were used: (1) patients who underwent pre-operative initial staging CT scans, and (2) were treated for rectal cancer with curative surgical intent. Exclusion criteria were (1) CT examination not performed at our centre, (2) staging scans of the chest not performed at the time of abdominopelvic scans and (3) missing initial CT staging scans. A total of 118 patients were identified of which 80 patients were eligible for analysis (Fig. 1).

All scans were performed at Western Health prior to any surgical or oncological intervention using a General Electric Lightspeed CT (GE Medical Systems, Milwaukee, WI) scanner and saved as Digital Imaging and Communications in Medicine (DICOM) images for analysis utilising Slice-O-Matic software (v.5.0 Tomovision, Montreal Canada). Standard CT procedures of 120 kV, 3 mm thickness, and a 512 × 512 matrix were used for all subjects. Two investigators (R.G. and A.A.) trained as per the Alberta protocol [8] completed landmarking and manual segmentation at the L3 level. Landmarking and segmentation was then progressed to T12 and T4 by investigator A.A. using single-slice axial CT images acquired at the midpoint of each of the fourth and twelfth thoracic vertebrae. Segmentation was undertaken highlighting muscle groups utilising anatomical knowledge and based upon existing protocols within the literature [6, 7]. The measurements at L3, T4 and T12 were validated by a second reader (R.G.) through random selection of images.

**Fig. 2:**
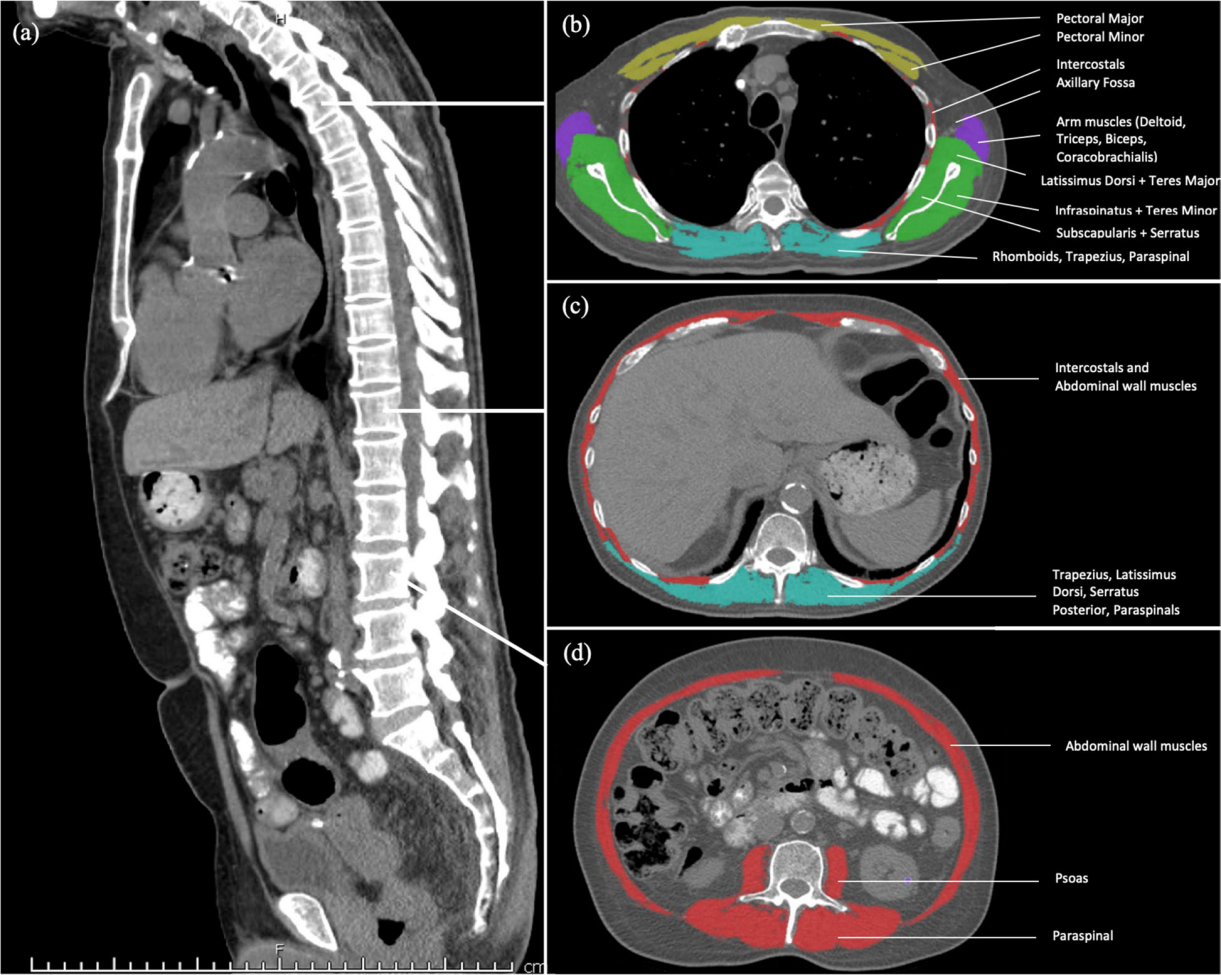
CT imaging illustrating an example of muscle composition at the T4, T12 and L3 levels, in a 73 year old male. **a** Sagittal CT image utilised to identify individual vertebral levels. **b** Transverse CT image at T4 highlighting the segmented total area (SMI. 32.9cm^2^/m^2^). **c** Transverse CT image at T12 highlighting the segmented total area (SMI 18.2cm^2^/m^2^). **d** Transverse CT image at L3 highlighting the segmented total area (SMI. 28.2cm^2^/m^2^)

Each abdominal and thoracic axial CT was segmented to distinguish the various muscle groups at the T4, T12 and L3 levels using anatomical knowledge and tissue-specific Hounsfield Unit (HU) ranges as highlighted in Fig. 2. Cross-sectional area (CSA (cm^2^)) of the sum of the muscles were computed for each image. Skeletal muscle (SM) cross-sectional areas (cm^2^) were calculated using standard Hounsfield Unit ranges [SM: -29 – 150]. The ranges were chosen based on previous recommendations [9]. The skeletal muscle index (SMI cm^2^/m^2^) was determined by normalising the muscle area for the patient’s height in meters squared, similar to body mass index (by use of the Mostellar formula) [10] i.e. CSA/(height (metres)^2^). Both readers were blinded to patient clinical status and outcomes.

In our study we utilised the Prado et al. defined sex-specific cut-offs for L3 vertebral level skeletal muscle index (SMI) associated with mortality ascertained by optimum stratification. These were 52.4 cm^2^/m^2^ for men and 38·5 cm^2^/m^2^ for women; patients below these values were classified as having sarcopenia [11].

### Statistical analysis

Descriptive statistics included mean, standard deviation and median, inter-quartile range (IQR) as appropriate. We compared SMI measurements at T4 and T12 against our reference at L3 by first computing a percentile for each ranked difference between T4 SMI and L3 SMI and between T12 SMI and L3 SMI. Folded empirical cumulative distribution curves (mountain plots) were generated: all percentiles over 50, percentile (y-axis) = 100-percentile were then plotted against differences (x-axis). The mountain plot provides information about the distribution of the differences [12]. As correlation metrics are not measures of agreement but only measures of linear association, analyses of measurement errors (T4 SMI and T12 SMI against a reference L3 SMI) were hence conducted using Ordinary Least Product (OLP) regression to uncover systematic differences and, in particular, to detect and distinguish fixed and proportional bias [13–16]. To quantify reliability of test–retest for duplicate measurements, we used the coefficient of repeatability (CR) [17, 18]. All tests were two-sided, and *p* < 0.050 was considered significant. Statistical analyses were performed using Systat v12 (Systat Software, Inc., Chicago, IL, USA) for OLP; MedCalc® Statistical Software version 20.116 (MedCalc Software Ltd, Ostend, Belgium) for folded empirical cumulative distribution plots and StatsDirect v. 3.0 (StatsDirect Ltd, Cheshire, UK) for coefficient of repeatability.

## Results

Of the total 80 patients included in the study, 21 (26%) were female and 59 (74%) male patients; mean age of 63.0 ± 13.0 (range, 30–86 years). The main baseline patient characteristics are summarised in Table 1. The mean CSA (cm^2^) were larger at the T4 level (165.3 cm^2^) compared to that at the T12 (94.4 cm^2^) and L3 levels (142.9 cm^2^), *p* < 0.001. The mean SMI (cm^2^/m^2^) was also larger at the T4 level (57.8 cm^2^/m^2^) compared to that at the T12 (33.1 cm^2^/m^2^) and L3 levels (49.9 cm^2^/m^2^), *p* < 0.001. Whilst females had a larger BMI with a mean of 30.6, as compared to males (26.8, *p* = 0.006), muscle area was significantly larger in men at all vertebral levels, *p* < 0.001 (Table 2). When we applied the Prado et al. definitions for thresholds for sarcopenia (< 52.4 cm^2^/m^2^ for males and < 38.5 cm^2^/m^2^ for females) [11] to our cohort, 37 patients (46%) had evidence of sarcopenia at the L3 vertebral level, with males exhibiting higher rates of sarcopenia (6 female vs 31 male).

**Table 1:** Characteristics of patients split by age (*n* = 80)

		65–74	< 65	≥ 75	*p*-value
N		20	43	17	
AGE		70.7 (2.9)	52.9 (8.2)	79.6 (2.9)	< 0.001
SEX	F	6 (30%)	10 (23%)	5 (29%)	0.81
	M	14 (70%)	33 (77%)	12 (71%)	
BMI		27.4 (4.8)	28.1 (6.4)	27.5 (4.4)	0.87
T4	CSA	149.9 (31.3)	180.2 (33.0)	145.9 (30.2)	< 0.001
	SMI	54.7 (12.8)	60.8 (9.7)	53.7 (8.4)	0.020
T12	CSA	87.2 (20.2)	99.8 (22.6)	89.1 (19.4)	0.054
	SMI	31.8 (8.0)	33.8 (7.3)	32.9 (6.3)	0.61
L3	CSA	130.0 (27.5)	151.4 (32.9)	136.4 (33.2)	0.033
	SMI,	47.3 (10.7)	51.0 (9.5)	50.0 (9.5)	0.37

Data are mean (SD) or n (%), *p*-value from t-test. *F* Female, *M* Males, *BMI* Body Mass Index, *CSA* Cross sectional area, *SMI* Skeletal Muscle Index, *T4* Fourth thoracic vertebral level, *T12* Twelfth thoracic vertebral level, *L3* Third lumbar vertebral level, *SD* Standard Deviation

**Table 2:** Body composition measures comparing females and males (*n* = 80)

	**Level**	**Female**	**Male**	***p*** **-value**
**N**		21	59	
**BMI**		30.6 (7.2)	26.8 (4.5)	0.006
**Muscle CSA**	**T4**	128.9 (21.1)	178.3 (30.1)	< 0.001
	**T12**	75.5 (12.5)	101.1 (20.1)	< 0.001
	**L3**	107.9 (18.2)	155.3 (27.3)	< 0.001
**SMI**	**T4**	51.3 (9.0)	60.1 (10.4)	< 0.001
	**T12**	30.1 (5.4)	34.2 (7.5)	0.026
	**L3**	42.8 (6.3)	52.4 (9.6)	< 0.001

Data are mean (SD) or n (%), *p*-value from t-test. *BMI* Body Mass Index, *CSA* Cross sectional area, *SMI* Skeletal Muscle Index, *T4* Fourth thoracic vertebral level, *T12* Twelfth thoracic vertebral level, *L3* Third lumbar vertebral level, *SD* Standard Deviation

**Fig. 3:**
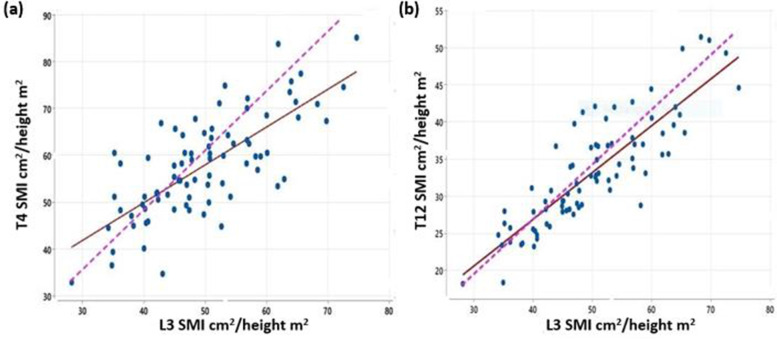
Scatterplots of thoracic level SMI against L3.** a** Scatter-plot of T4 SMI against L3 SMI; **b** T12 SMI against L3 SMI.  line of best fit;  line of equality

**Fig. 4:**
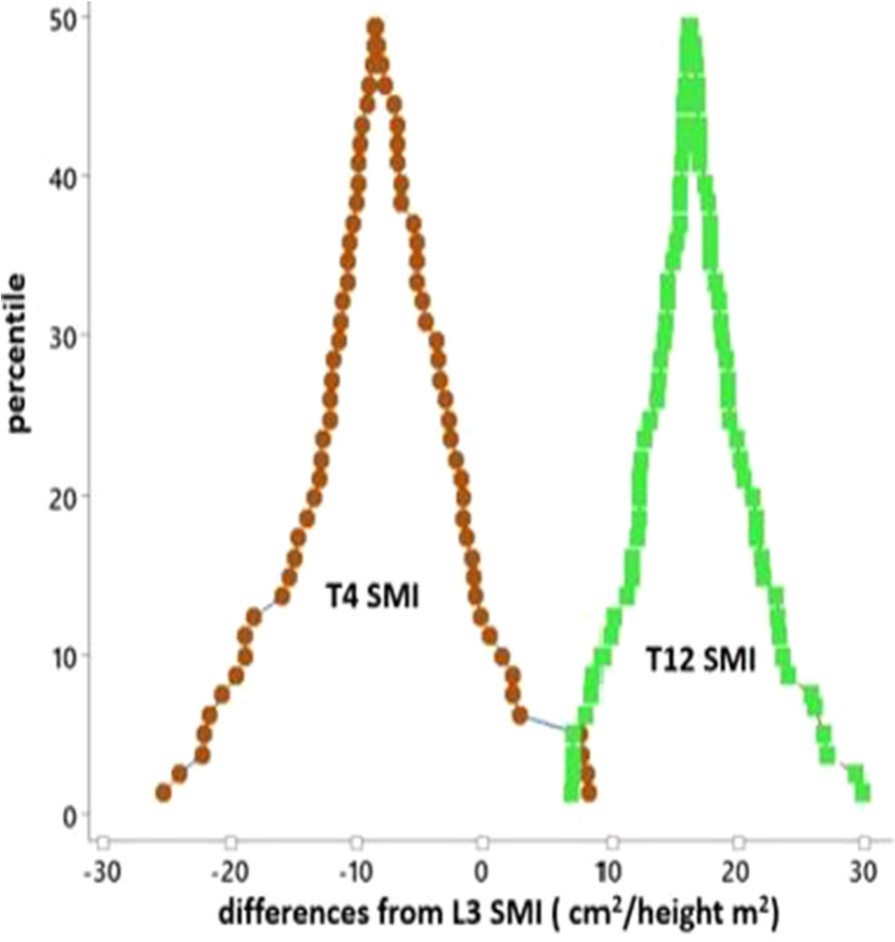
Folded empirical cumulative distribution curves

The scatter diagrams in Fig. 3 show that both the SMI at T4 and T12 had a linear relationship with measurements from L3. The mountain plots in Fig. 4 demonstrate a larger distribution of differences between T12 and L3 than that of T4 and L3. OLP regression were then conducted which shows no fixed or proportional bias between T4 and L3, whilst when T12 was analysed against L3 there was evidence of a proportional bias (Table 3).

**Table 3:** Ordinary least product (OLP) regression to detect fix and proportional bias

**(a) T4 SMI vs L3 SMI**				
**Parameter**	**Estimate**	**ASE**	**Parameter/ASE**	**Wald 95% CI interval**
intercept	3.271	4.550	0.719	-5.788 to 12.311
slope	1.093	0.090	12.206	0.195 to 1.272
**(b) T12 SMI vs L3 SMI**				
**Parameter**	**Estimate**	**ASE**	**Parameter/ASE**	**Wald 95% CI interval**
intercept	-3.695	2.273	-1.626	-8.220 to 0.830
slope	0.738	0.045	16.489	0.649 to 0.827

T4 *vs* L3: no fix or proportional bias; T12 *vs* L3: proportional bias detected. *ASE* asymptotic standard error, *CI* confidence interval

For the interobserver validation of our measurement techniques, readers (A.A.), and (R.G.) independently evaluated five selected images (chosen randomly) at each of the levels of T4, T12 and L3. Duplicate measurements were quantified using the Coefficient of Repeatability (CR), the value below which the absolute differences between two measurements would lie with a probability of 95%. The presence of a larger variability for the CSA between the L3 T12 levels (76.00) compared to that comparing L3-T4 (34.10), implied that T4 CSA measurements had higher test–retest reliability (Table 4).

**Table 4:** Coefficient of repeatability (CR cm^2^) for duplicate measurements

**Muscle CSA (cm**^**2**^ **)**	**L3 ** ***minus*** ** T4**	**L3 ** ***minus*** ** T12**
Within-subjects SD	12.3019	27.4193
CR (for alpha 0.05)	34.10	76.00

The lower the CR, the higher the test–retest reliability. *CSA* cross-sectional area, *SD* standard deviation

**Fig. 5:**
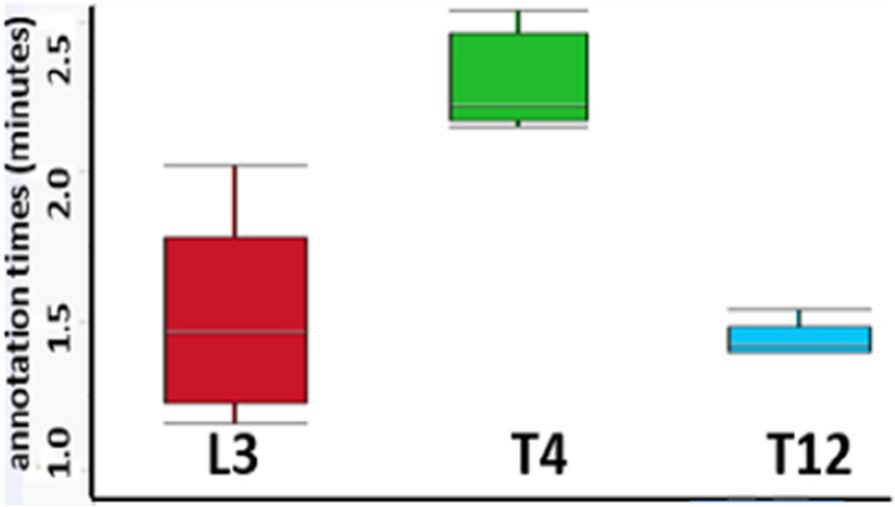
Difference in annotation time between L3 vs T4 and T12 regions

Two independent readers (A.A.) and (J.Y.) measured the timing needed for annotating the skeletal muscle of five randomly selected CT images at each level. we demonstrated that there was a statistically significant difference between the time required to measure skeletal muscle mass at vertebral levels T4 compared with L3, favouring T12 with regards to time for measurement (median difference between L3 and T4: 0.85 min, *p* = 0.009). In contrast, the time to measure L3 and T12 (-0.03 min; *p* = 0.75) was similar (Fig. 5).

Although various cut-offs have been described within the literature, there is only a very limited number of studies available and none have been widely validated at both the T4 and T12 levels [5, 19]. As there were no studies comparing vertebral thoracic levels T4, T12 and L3 in a colorectal population, we therefore divided our patients into four groups according to SMI, allowing us to visualise the effects of both the highest and lowest quartile SMI. This method has was similar to how Lee et al. analysed their data in their study comparing T4 with that of L3 in pre-operative patients undergoing lung transplantation [6].

Sarcopenia was assessed by analysing the SMI from CT images at the T4, T12 and L3 and was defined by the lowest quartile of SMI (Q1). Sarcopenia based on this definition generated the following cut-off values; T4 SMI < 50.47, T12 SMI < 28.01, L3 SMI < 42.36. Using this information, we then assessed the association between this “newly defined” L3 sarcopenia definition with the established Prado et al. [11] definition. We demonstrated that there was a statistically significant relationship between these two definitions (chi-square with one degree of freedom = 6.051, with *p* = 0.014). Whilst the comparison between sarcopenic patients defined at T12 and those defined by Prado were statistically significant (*P* < 0.001), those comparisons at T4 showed no significance (*P* = 0.052).

We then investigated whether sarcopenia affected outcomes including medical and surgical complications, development of locoregional recurrence and distal metastases, as well as total hospital length of stay (LOS) and death. Whilst the sarcopenic patients (Q1) showed poorer survival at all three vertebral levels (T4: *P* = 0.003; T12: *P* = 0.013; L3: *P* = 0.013), there was no association with medical and surgical complications, recurrence rates nor length of stay (Table 5).

**Table 5:** Post-operative outcomes in sarcopenic vs non-sarcopenic patients using new definition based on SMI quartile cut-offs

		**T4**			**T12**			**L3**		
		**Q1**	**Q4**	***p*** **-value**	**Q1**	**Q4**	***p*** **-value**	**Q1**	**Q4**	***p*** **-value**
**N**		20			20			20		
**Medical Complications**	No	11 (55%)	14 (70%)	0.11	10 (50%)	15 (75%)	0.1	10 (50%)	13 (65%)	0.34
	Yes	9 (45%)	6 (30%)		10 (50%)	5 (25%)		10 (50%)	7 (35%)	
**Surgical Complications**	No	13 (65%)	14 (70%)	0.74	14 (70%)	15 (75%)	0.72	15 (75%)	12 (60%)	0.31
	Yes	7 (35%)	6 (30%)		6 (30%)	5 (25%)		5 (25%)	8 (40%)	
**Recurrence**	No	2 (67%)	2 (50%)	0.66	3 (60%)	3 (75%)	0.64	3 (60%)	4 (100%)	0.15
	Yes	1 (33%)	2 (50%)		2 (40%)	1 (35%)		2 (40%)	0 (0%)	
**Death**	No	11 (55%)	19 (95%)	0.003	11 (55%)	18 (90%)	0.013	11 (55%)	18 (80%)	0.013
	Yes	9 (45%)	1 (5%)		9 (45%)	2 (10%)		9 (45%)	2 (10%)	
**LOS days, median (IQR)**		11 (8.5, 19)	9 (7, 10.5)	0.062	10 (7.5, 13.5)	9 (7.5, 10)	0.29	10.5 (8.5, 16.5)	9 (7, 11)	0.23

Data are n (%), *p*-value from Pearson’s chi-squared test

## Discussion

This study has shown that CT derived muscle mass at T4 is comparable to those measures obtained from the L3 level in patients with rectal cancer. Body composition measurements at T12 revealed a larger distribution of differences and a proportional bias was noted (Fig. 4, Table 3).

In method comparison studies, the appropriate analysis should aim to uncover systematic differences. There are two potential sources of systematic disagreement between methods of measurement: fixed and proportional bias. Fixed bias means that one method gives values that are higher (or lower) than those from the other by a constant amount; proportional bias means that one method gives values that are higher (or lower) than those from the other by an amount that is proportional to the level of the measured variable [13–16].

Previous studies on T4 and T12 CSA [5, 7, 20] have reported findings contradictory to our own results. High values of Pearson’s correlation coefficients (r) had been used as metrics to assess agreements with L3 measurements in other publications [21, 22]. However, it has been well documented in the statistical literature that Pearson’s correlation coefficient merely indicates the scatter of values around the line of best fit, (Fig. 3) regardless of whether the slope of that line differs from unity (proportional bias) or whether its intercept differs from zero (fixed bias) [13–16]. It does no more than indicate the strength of the linear association between the x and y variables in the examined population. The information provided by r is, therefore, of no value in detecting systematic biases between methods. Ordinary least product (OLP) regression and or Bland–Altman plot [21, 22] are the appropriate analysis to use in these situations.

Utilising CT derived muscle mass at the L3 is currently the gold standard for body composition analysis, which serves a marker for total body skeletal muscle quantity [9, 10, 23, 24]. Although artificial intelligence (AI) derived 3D total body composition measurements have been developed and have been described within the literature, there are very few studies using validated patient data, and most clinical data linked research is generally only based on single level semiautomated assessments [4]. There is also growing evidence that other levels including T4 and T12 are equally representative of patient body composition, as shown in conditions such as lung cancers [5, 25], and patients undergoing interventional cardiothoracic and vascular surgery [19, 26].

In our study, we found that both total muscle cross sectional area (CSA) and skeletal muscle index (SMI) were significantly higher in males than in females; however female patients had significantly higher BMI compared to our male patients. We found that muscle measurement at all vertebral levels was relatively easy to complete by manual segmentation. When interobserver agreement was compared, the much larger variability seen for L3-T12 CSA compared to L3-T4 CSA implied that T4 CSA measurements had higher test–retest reliability. When we looked at time and consistency to complete body composition analysis between T4, T12 and L3 levels, we found that overall, the T12 vertebra reading required slightly less time to annotate compared to L3. However, the readings at T4 vertebral levels had a more consistent segmentation result between graders.

We also performed a comparison of the degree of muscle quantity between our thoracic and lumbar levels within our patient cohort. We found that the cross-sectional muscle areas were greatest at the vertebral T4 level, followed by L3 then finally T12. Comparing methods of measurement utilising percentile ranked differences and ordinary least product regression, we found a stronger agreement between both CSA and SMI measured at L3 with that at T4 as compared with T12. However, despite these agreements, we noticed that when trying to annotate the muscle groups within the T4 level, some of the patients’ skeletal muscles were “cut off” from the CT scan thereby affecting our ability to complete the total calculation of body composition in those images.

With regards to clinical outcomes, our study did identify evidence of sarcopenia within our cancer cohort; however, we did not find an association between sarcopenia and post-operative complications, recurrence rates or hospital LOS in patients undergoing curative resection. We did however identify that sarcopenia was related to a reduced survival.

The strength of this study is the robust analysis of measurement errors (fixed and proportional bias) as distinct from using correlation coefficients to assess agreements. We recognised that Pearson’s (r) value does not provide clinicians with any insight into systematic errors that may be inherent in the measurement obtained with a specific assessment tool. We therefore utilised ordinary least product (OLP) regression for our analysis.

There were several limitations to our work. Our study relied on retrospective data from a single-centre and only 5 scans at each vertebral level were read to determine the interobserver reliability. Whilst our study was able to determine a positive relationship between both T4 and T12 vertebral levels with L3, half of the images (40 out of 80) acquired at T4 were noted to have “cut offs”, where the outer circumference of the muscle mass was missing; a problem also seen in other studies assessing skeletal muscle at the T4 level [5, 27]. To account for this “cut off”, the “arm” muscles were not counted in the final cross-sectional area. This therefore led to a poorer reproducibility as it was not clear as to where the boundaries of the muscles around the scapula and arm might be. Previous studies encountered a similar problem and overcame this by including only the pectorals, intercostals and muscles of the back in their CSA measurements [27, 28].

The findings of a strong association between T4 and L3 measurements suggested that the thoracic muscles, like those at the lumbar level, would be reasonably representative of total body skeletal muscle quantity. However, consideration must be given to the functions of the muscles at their respective levels. For example, at the T12 and L3 levels, muscles include the rectus abdominis, external and internal oblique and erector spinae (the core muscles). These muscles are thought to initiate full-body functional movement and are essential for stabilising the body in dynamic movements [5]. Although some of these muscles (erector spinae) also extend upwards, the major muscles annotated at the T4 level, (pectoralis muscles, and supra and infraspinatus muscles) primarily function in a different way, through mobilisation of the arm and shoulder gridles, resulting in potentially different CSA and SMI results [5, 29].

## Conclusions

This study demonstrated that quantifying skeletal muscle mass at the T4 vertebral level is comparable to measures achieved at L3 in patients with rectal cancer, notwithstanding that annotation time for T4 measurements are longer. This new information may be useful in the future to allow clinicians to accurately compare the effect of body composition between different cancers using thoracic levels.

## Data Availability

We prefer not to share our patient raw data. However, the datasets used and/or analysed during the current study are available from the corresponding author on reasonable request.

## Abbreviations

ACCORD: Australian Comprehensive Cancer Outcomes and Research Database
AI: Artificial intelligence
BMI: Body mass index
CR: Coefficient of repeatability
CRC: Colorectal cancer
CT: Computed tomography
CSA: Cross sectional area
IQR: Inter-quartile range
LOS: Length of stay
OLP: Ordinary least product regression
SM: Skeletal muscle
SMI: Skeletal muscle index

## References

[1] Kawaguchi Y, Hanaoka J, Ohshio Y, Okamoto K, Kaku R, Hayashi K (2019). Sarcopenia predicts poor postoperative outcome in elderly patients with lung cancer. Gen Thorac Cardiovasc Surg.

[2] Rier HN, Jager A, Sleijfer S, van Rosmalen J, Kock M, Levin MD (2017). Low muscle attenuation is a prognostic factor for survival in metastatic breast cancer patients treated with first line palliative chemotherapy. Breast.

[3] van Dijk DP, Bakens MJ, Coolsen MM, Rensen SS, van Dam RM, Bours MJ (2017). Low skeletal muscle radiation attenuation and visceral adiposity are associated with overall survival and surgical site infections in patients with pancreatic cancer. J Cachexia Sarcopenia Muscle.

[4] Tolonen A, Pakarinen T, Sassi A, Kyttä J, Cancino W, Rinta-Kiikka I (2021). Methodology, clinical applications, and future directions of body composition analysis using computed tomography (CT) images: A review. Eur J Radiol.

[5] Grønberg BH, Sjøblom B, Wentzel-Larsen T, Baracos VE, Hjermstad MJ, Aass N (2019). A comparison of CT based measures of skeletal muscle mass and density from the Th4 and L3 levels in patients with advanced non-small-cell lung cancer. Eur J Clin Nutr.

[6] Lee S, Paik HC, Haam SJ, Lee CY, Nam KS, Jung HS (2016). Sarcopenia of thoracic muscle mass is not a risk factor for survival in lung transplant recipients. J Thorac Dis.

[7] Derstine BA, Holcombe SA, Ross BE, Wang NC, Su GL, Wang SC (2018). Skeletal muscle cutoff values for sarcopenia diagnosis using T10 to L5 measurements in a healthy US population. Sci Rep.

[8] TomoVision. sliceOmatic Alberta Protocol February 2017. Available from: https://tomovision.com/Sarcopenia_Help/index.htm.

[9] Mitsiopoulos N, Baumgartner RN, Heymsfield SB, Lyons W, Gallagher D, Ross R (1998). Cadaver validation of skeletal muscle measurement by magnetic resonance imaging and computerized tomography. J Appl Physiol (1985).

[10] Shen W, Punyanitya M, Wang Z, Gallagher D, St-Onge MP, Albu J (2004). Total body skeletal muscle and adipose tissue volumes: estimation from a single abdominal cross-sectional image. J Appl Physiol (1985).

[11] Prado CMM, Lieffers JR, McCargar LJ, Reiman T, Sawyer MB, Martin L (2008). Prevalence and clinical implications of sarcopenic obesity in patients with solid tumours of the respiratory and gastrointestinal tracts: a population-based study. Lancet Oncol.

[12] Monti KL (1995). Folded empirical distribution function curves—mountain plots. Am Stat.

[13] Ludbrook J (2010). Confidence in Altman-Bland plots: a critical review of the method of differences. Clin Exp Pharmacol Physiol.

[14] Ludbrook J (1997). Comparing methods of measurement. Clin Exp Pharmacol Physiol.

[15] Ludbrook J (2010). Linear regression analysis for comparing two measurers or methods of measurement: but which regression?. Clin Exp Pharmacol Physiol.

[16] Ludbrook J (2012). A primer for biomedical scientists on how to execute model II linear regression analysis. Clin Exp Pharmacol Physiol.

[17] Vaz S, Falkmer T, Passmore AE, Parsons R, Andreou P (2013). The case for using the repeatability coefficient when calculating test–retest reliability. PLoS One.

[18] Bland JM, Altman DG (1996). Statistics notes: measurement error. BMJ.

[19] Panthofer AM, Olson SL, Harris DG, Matsumura JS (2019). Derivation and validation of thoracic sarcopenia assessment in patients undergoing thoracic endovascular aortic repair. J Vasc Surg.

[20] Phan EN, Thorpe SW, Wong FS, Saiz AM, Taylor SL, Canter RJ (2020). Opportunistic muscle measurements on staging chest CT for extremity and truncal soft tissue sarcoma are associated with survival. J Surg Oncol.

[21] Altman DG, Bland JM (1983). Measurement in medicine: the analysis of method comparison studies. J R Stat Soc Ser D (The Statistician).

[22] Bland JM, Altman DG (1996). Measurement error and correlation coefficients. BMJ.

[23] Fearon K, Strasser F, Anker SD, Bosaeus I, Bruera E, Fainsinger RL (2011). Definition and classification of cancer cachexia: an international consensus. Lancet Oncol.

[24] Bahat G, Turkmen BO, Aliyev S, Catikkas NM, Bakir B, Karan MA. Cut-off values of skeletal muscle index and psoas muscle index at L3 vertebra level by computerized tomography to assess low muscle mass. Clin Nutr. 2021;40(6):4360–5. 10.1016/j.clnu.2021.01.010 33516603

[25] Wysham NG, Nipp RD, LeBlanc TW, Wolf SP, Ekstrom MP, Currow DC (2016). A practical measurement of thoracic sarcopenia: correlation with clinical parameters and outcomes in advanced lung cancer. ERJ Open Res.

[26] Nemec U, Heidinger B, Sokas C, Chu L, Eisenberg RL (2017). Diagnosing Sarcopenia on Thoracic Computed Tomography: Quantitative Assessment of Skeletal Muscle Mass in Patients Undergoing Transcatheter Aortic Valve Replacement. Acad Radiol.

[27] van Heusden HC, Swartz JE, Chargi N, de Jong PA, van Baal M, Wegner I (2021). Feasibility of assessment of skeletal muscle mass on a single cross-sectional image at the level of the fourth thoracic vertebra. Eur J Radiol.

[28] Nishimura JM, Ansari AZ, D'Souza DM, Moffatt-Bruce SD, Merritt RE, Kneuertz PJ (2019). Computed Tomography-Assessed Skeletal Muscle Mass as a Predictor of Outcomes in Lung Cancer Surgery. Ann Thorac Surg.

[29] Anderson DE, D'Agostino JM, Bruno AG, Demissie S, Kiel DP, Bouxsein ML (2013). Variations of CT-based trunk muscle attenuation by age, sex, and specific muscle. J Gerontol A Biol Sci Med Sci.

